# (In)Distinctive Role of Long Non-Coding RNAs in Common and Rare Ovarian Cancers

**DOI:** 10.3390/cancers13205040

**Published:** 2021-10-09

**Authors:** Maja Sabol, Jean Calleja-Agius, Riccardo Di Fiore, Sherif Suleiman, Sureyya Ozcan, Mark P. Ward, Petar Ozretić

**Affiliations:** 1 Laboratory for Hereditary Cancer, Division of Molecular Medicine, Ruđer Bošković Institute, HR-10000 Zagreb, Croatia; maja.sabol@irb.hr; 2 Department of Anatomy, Faculty of Medicine and Surgery, University of Malta, MSD 2080 Msida, Malta; jean.calleja-agius@um.edu.mt (J.C.-A.); riccardo.difiore@um.edu.mt (R.D.F.); sherif.s.suleiman@um.edu.mt (S.S.); 3 Sbarro Institute for Cancer Research and Molecular Medicine, Center for Biotechnology, College of Science and Technology, Temple University, Philadelphia, PA 19122, USA; 4 Department of Chemistry, Middle East Technical University (METU), 06800 Ankara, Turkey; sozcan@metu.edu.tr; 5 Cancer Systems Biology Laboratory (CanSyl), Middle East Technical University (METU), 06800 Ankara, Turkey; 6 Department of Histopathology, Trinity St James’s Cancer Institute, Emer Casey Molecular Pathology Laboratory, Trinity College Dublin and Coombe Women’s and Infants University Hospital, D08 RX0X Dublin, Ireland; wardm6@tcd.ie

**Keywords:** rare ovarian cancers, long non-coding RNAs, lncRNAs, competing endogenous RNA, ceRNA, MALAT1, ovarian cancer biomarkers, lncRNA-based therapy

## Abstract

**Simple Summary:**

Ovarian cancers (OCs) are the most lethal form of gynecological tumors. The commonest are high-grade serous OCs, while rare OCs originate from many different cell types, such as epithelial, germ cell, sex cord-stromal, or mixed types. Rare OCs have distinct molecular characteristics, prognosis, and therapeutic approaches. However, all ovarian malignancies mostly share the same problem: late diagnosis due to the lack of specific symptoms. Therefore, there is a perpetual need to discover better diagnostic, prognostic, and predictive biomarkers, as well as new therapeutic approaches. In recent years, long non-coding RNAs (lncRNAs) have gained widespread attention because of their important role in various biological pathways. They have multiple mechanisms of action with an important role in many cellular processes related to OCs development and progression. This review will focus on the different aspects of lncRNAs in OCs and attempt to highlight the distinctive role of lncRNAs in common and rare OCs.

**Abstract:**

Rare ovarian cancers (ROCs) are OCs with an annual incidence of fewer than 6 cases per 100,000 women. They affect women of all ages, but due to their low incidence and the potential clinical inexperience in management, there can be a delay in diagnosis, leading to a poor prognosis. The underlying causes for these tumors are varied, but generally, the tumors arise due to alterations in gene/protein expression in cellular processes that regulate normal proliferation and its checkpoints. Dysregulation of the cellular processes that lead to cancer includes gene mutations, epimutations, non-coding RNA (ncRNA) regulation, posttranscriptional and posttranslational modifications. Long non-coding RNA (lncRNA) are defined as transcribed RNA molecules, more than 200 nucleotides in length which are not translated into proteins. They regulate gene expression through several mechanisms and therefore add another level of complexity to the regulatory mechanisms affecting tumor development. Since few studies have been performed on ROCs, in this review we summarize the mechanisms of action of lncRNA in OC, with an emphasis on ROCs.

## 1. Introduction

Gynecologic malignancies comprise about 19% of all cancers diagnosed in women, and out of these more than 50% are classified as rare [1]. They encompass more than 30 different histologic diagnoses of various sites, such as vulva, vagina, uterine cervix and corpus, fallopian tube, and ovary. Ovarian cancers (OCs) are the deadliest of all gynecological malignancies, and in 2020 there were around 314,000 new cases and 207,000 deaths related to them around the globe [2]. They mostly fall into the epithelial subtype based on the cell of origin, and the most malignant but also the most common type is the high-grade serous OC (HGSOC) [3]. Rare cancers are usually defined as malignancies that have an incidence of <6 cases per 100,000 [4]. Rare ovarian cancers (ROCs) are malignancies of the ovary which can originate from different cell types, such as epithelial, germ cell, sex cord-stromal, or mixed types [5]. Ovarian malignancies mostly share the same problem, which is late diagnosis [6]. ROC malignancies lack distinguishable symptoms during the early stages of disease development, which means that most cancers are diagnosed late when they have already progressed to advanced stages of the disease and are therefore more difficult to treat. This is especially true for ROCs, as they can be misdiagnosed due to their rarity and clinical inexperience. This results in poor outcomes for these patients and stresses the need for reliable markers for early diagnosis and potential specific targets for therapy. In recent years, one class of macromolecules called long non-coding RNAs (lncRNAs) has been emerging as promising diagnostic and prognostic biomarkers, as well as potential therapeutic targets, for many different types of cancers [7]. Although the role of lncRNAs in OCs has been extensively reviewed previously [8,9,10,11,12,13,14,15], this is, to the best of our knowledge, the first effort to differentiate the role of lncRNAs between common and rare OCs.

Generally, a major drawback in the research on ROCs is the lack of studies that separate the OC subtypes, as in most cases the samples are just classified as OC. Most studies examine OC in general or focus on the most prevalent HGSOC subtype. Even LGSOC is not well characterized within these studies, which makes analyzing available data difficult, and possibly misleading. Therefore, this review will outline the lncRNAs associated with OC in general, with the OC subtype defined where available, focusing on ROCs when possible.

## 2. Common Versus Rare Ovarian Cancers

OC is a major cause of morbidity and mortality in women, with minimal improvement in survival rates over the past decades [16]. OC is divided into epithelial and non-epithelial subgroups. However, OC is a heterogeneous malignancy with diverse pathophysiology and clinical development. In the case of epithelial ovarian cancers (EOCs), the majority of tumors originate outside the ovary, while only a subset develops within the ovarian surface epithelium [17]. Approximately 90% of OCs belong to the malignant epithelial tumor (carcinomas) group, and there are currently five main subtypes of carcinoma: high-grade serous ovarian carcinoma (HGSOC), low-grade serous ovarian carcinoma (LGSOC), endometrioid carcinoma; clear cell carcinoma, and mucinous carcinoma [18]. HGSOC and endometrioid cancers are considered common OCs, while the others are deemed to be rare. Table 1 summarizes the most common molecular alterations as well as the prognosis of different types of ovarian cancers.

HGSOCs are chromosomally unstable tumors that commonly have mutations in the *TP53* tumor suppressor gene [19]. In most cases, there are also germline or somatic mutations in *BRCA1* or *BRCA2*, or hypermethylation of *BRCA1* promoter with a loss of expression. The underlying loss of BRCA1/2 function and inability to repair double-strand repair breaks, which in turn leads to chromosomal instability, lead to a potential role for drugs targeting the DNA repair (e.g., PARP inhibitors) [20]. The PI3K-AKT pathway plays an important role in HGSOC [3]. The 5-year overall survival rates are 84.0%, 67.7%, and 32.1% for stage IA/IB, IC/II, and III/IV HGSOC, respectively [21].

**Table 1:** Molecular alterations and prognosis of different types of ovarian cancers.

Cancer Type	Common or Rare	Molecular Alterations	Altered Pathways	Prognosis	Reference
**Epithelial**					
High-Grade Serous Ovarian Carcinoma (HGSCO)	Common	Genomic Instability, *BRCA1*, *BRCA2*, *TP53*,	PI3K-AKT	Almost 30% of Patients Die within 5-years of Diagnosis	[3,19,20]
Low-Grade Serous Ovarian Carcinoma (LGSOC)	Rare	*KRAS, BRAF, ERBB2, PIK3CA, FFAR1, USP9X* and *EIF1AX*	MAPK and AKT-mTOR	Better than The High-Grade Serous Cancer	[22]
Endometrioid Carcinoma (EC)	Common	*CTNNB1*, *CDKN2A*, *PIK3CA*, *KRAS*, *ARID1A*, *PTEN*, and *PPP2R1A*	WNT, MAPK/RAS and PI3K	Good	[19,20]
Ovarian Clear Cell Carcinoma (OCCC)	Rare	*ARI1D1A*, *PI3KCA*, *PPP2R1A* and *KRAS*	PI3K and mTOR	Favorable Compared with The Serous Cancer	[23,24,25]
Mucinous ovarian carcinoma (MOC)	Rare	High Microsatellite Instability (MSI-H), *KRAS*, *CTNNB1,* or *APC*	WNT	Worse than Advanced Stage Serous Cancer	[23,26,27,28]
**Non-Epithelial**					
Sex-Cord Stromal Tumors (Granulosa Cell Tumors)	Rare	*FOXL2*	PI3K/AKT, TGF-β, and Notch	Good	[29,30,31,32]
Sex-Cord Stromal Tumors (Sertoli–Leydig Cell Tumors)	Rare	*DICER1, FOXL2*	Unknown	Good	[33]
Germ-Cell Tumors	Rare	*KIT*, *KRAS*	Unknown	Unknown	[34]

Endometrioid carcinomas (EC) are the second commonest malignant ovarian neoplasm, accounting for 8–15% of all ovarian carcinomas. There is a strong association with endometriosis. Most EC is grade 1 or 2. Since at presentation they are predominantly low stage and low grade, the burden of morbidity and mortality associated with this subtype is relatively low, despite being common. The 5-year overall survival rates are 87.1%, 83.9%, and 44.7% for stage IA/IB, IC/II, and III/IV EC, respectively [21]. There are no data currently available specifically on the benefit of adjuvant chemotherapy in patients with advanced-stage EC, and such data will be hard to acquire given the rarity of advanced-stage or recurrent tumors. Genes that are typically mutated in EC include *CTNNB1*, *CDKN2A*, *PIK3CA*, *KRAS*, *ARID1A*, *PTEN*, and *PPP2R1A* [20]. Moreover, the MAPK/RAS, WNT, and PI3K pathways could be good candidate targets for molecular therapeutics [35].

LGSOC represent <5% of all ovarian serous carcinomas, affecting younger women with a median age of under 55 years. The 5-year overall survival rates are 93.2%, 82.7%, and 54.2% for stage IA/IB, IC/II, and III/IV LGSOC, respectively [21]. Despite its slow growth, there is a poor sensitivity of LGSOC to chemotherapy. In this type of malignancy, there are mutations of genes involved in the mitogen-activated protein kinase (MAPK) pathway, such as *BRAF*, *KRAS*, *NRAS,* and *ERBB2*, but occasionally there may be driver mutations of *FFAR1*, *PIK3CA*, *USP9X,* and *EIF1AX* linked to the AKT-mTOR pathway [22]. Moreover, RAD50 and NBS1 proteins are absent in LGSOC. Since estrogen and progesterone receptors are often expressed in LGSOC, hormone therapy can be a potential therapeutic alternative. Targeted therapy such as trametinib, a mitogen-activated protein kinase inhibitor, could represent a new standard of care treatment option for women with recurrent LGSOC.

Ovarian clear cell carcinoma (OCCC) accounts for approximately 5% of all ovarian carcinomas. When diagnosed at an earlier stage, it tends to have a good prognosis as surgery is often curative. In advanced stages, however, it is associated with a poor prognosis as it is often chemoresistant [23]. The 5-year overall survival rates are 81.7%, 69.0%, and 22.3% for stage IA/IB, IC/II, and III/IV OCCC, respectively [21]. OCCC and clear-cell carcinoma of the kidneys share similar molecular pathways [24], and therefore targeted therapy aiming to inhibit angiogenesis, growth-factor signaling, and mTOR pathways might improve prognosis. Although preliminary clinical data focusing only on OCCC are limited, treatment with multikinase inhibitors (axitinib, sunitinib, sorafenib, and pazopanib), bevacizumab, temsirolimus, and everolimus may have anti-tumor activity in this particular malignancy. In OCCC, the most common somatic genetic alterations are loss of *ARID1A*, activation of PIK3CA, and mutations in *PPP2R1A* and *KRAS* [25]. Novel treatment strategies in the case of *ARID1A* mutated OCCC include the inhibition of the methyltransferase EZH2 and the administration of dasatinib and/or the HDAC6 inhibitor ACY1215. Since EGFR expression is detected in up to 60% of OCCC cases, EGFR inhibitors may also be effective. In addition, mTOR inhibitors may also be promising due to the high expression of mTOR in both early- and advanced-stage OCCC [27].

Mucinous epithelial ovarian cancer (MOC) accounts for less than 5% of EOCs. The 5-year overall survival rates are 82.9%, 69.,5%, and 13.9% for stage IA/IB, IC/II, and III/IV OCCC, respectively [21]. While it is recommended to treat MOC with adjuvant carboplatin and paclitaxel, since HER2 is amplified or expressed in up to 19% of cases, trastuzumab and HER2- targeted therapies might be an effective treatment [23,26]. Molecular alterations in MOCs commonly involve *KRAS*, as well as mutations in *CTNNB1* or *APC* gene, and high microsatellite instability (MSI-H) [28].

Sex-cord stromal tumors and malignant germ-cell tumors are very rare non-epithelial ovarian tumors, which overall account for only 6% of all ovarian malignancies [36,37,38,39]. A common type of malignant sex-cord stromal tumor is granulosa cell tumor (GCT), of which there are the adult type and juvenile granulosa cell tumors. Granulosa cell tumors secrete progesterone and estrogen [29]. Testing for the *FOXL2* c.402C > G (p.C134W) mutation is helpful in the diagnosis of adult-type tumors [30]. However, GATA4, SMAD, VEGF, PI3K/AKT, AMH, and TGF-β are also involved in this type of cancer [31]. Granulosa cell tumors tend to have a slow progression and late recurrence. In the case of women with advanced-stage or recurrent granulosa cell tumors, traditional chemotherapy is limited in effectiveness [40]. Carboplatin and paclitaxel, a combination of bleomycin, etoposide, and cisplatin (BEP), and targeted therapies including VEGF inhibitors, tyrosine kinase inhibitors (TKIs), and hormonal treatment have been investigated as possible therapeutic options [23]. For Sertoli–Leydig cell tumors, *DICER1* mutations seem to be a prognostic factor that is associated with a higher risk of relapse, especially in younger patients. In these tumors, adjuvant chemotherapy is recommended in stage IA disease particularly if there is poor differentiation or heterologous elements. BEP is commonly used, or else etoposide/cisplatin, carboplatin, and paclitaxel, cyclophosphamide/doxorubicin/cisplatin, or platinum agents alone. The 5-year overall survival rates are 90.7% and 76.2% for granulosa cell tumors and Sertoli-Leydig cell tumors, respectively [41].

Germ-cell tumors can be histologically classified as dysgerminoma, immature teratoma, yolk sac tumor, choriocarcinoma, embryonal carcinoma, mixed germ-cell tumor, malignant struma ovarii, gonadoblastoma, and teratoma with malignant transformation [18]. They are different from EOCs because they tend to be diagnosed in younger women, and due to their rapid tumor growth, they are usually symptomatic, thus leading to earlier diagnosis and a significantly better prognosis. Germ-cell tumors also have a different set of biomarkers such as alpha-fetoprotein (AFP), serum human chorionic gonadotropin (HCG), and lactate dehydrogenase (LDH) [42]. The most common mutations were found in *KIT* and *KRAS*, akin to testicular germ cell tumors [34]. Platinum-based regimens have led to a 5-year overall survival of over 90% for early-stage tumors and above 75% for advanced disease [37]. Adjuvant chemotherapy such as BEP is being used routinely. In the case of relapsed ovarian germ-cell tumors, treatment includes the use of paclitaxel, ifosfamide, and cisplatin, with extrapolation from current treatment used for testicular germ-cell tumors, and even using more complex regimens containing combinations of methotrexate, cisplatin, vincristine, and bleomycin, alternating with cyclophosphamide, etoposide and actinomycin D [36]. Furthermore, targeted therapies that have been investigated consist of trastuzumab (anti-HER2 monoclonal antibody, tyrosine kinase inhibitors (TKIs) (i.e., imatinib and sunitinib), and antiangiogenic agents such as thalidomide and bevacizumab [29].

In contrast to almost all other common OCs, the treatment of ROCs is challenging and often must be based on expert opinion, retrospective studies, or extrapolation from other tumor sites with similar histology. This leads to difficulty in developing effective guidelines for clinical practice.

### Availability of Cell Culture Models for Studying Common and Rare Ovarian Cancers

Cancer and its corresponding healthy tissue cell lines are indispensable in vitro models for discovering new biomarkers and pre-clinical drug testing. Due to its extreme heterogeneity, there are many problems with using OCs cell lines as models for so many different OCs subtypes. One of the biggest problems is often misclassification of commonly used cell lines with respect to their tumor subtype [43]. Domcke et al., analyzed a panel of 47 commonly used OC cell lines and found significant genetic differences between them and HGSOC tumor tissue samples, while several less frequently used cell lines were found to be more genetically alike to the primary tumors [44]. A collection of 25 rigorously validated OC cell lines was reported to properly represent the OC subtypes from which they are derived [45]. Just recently, Barnes et al., used non-negative matrix factorization on transcriptomic data of 44 EOC cell lines to properly classify them into the five major histologically distinct subtypes (HGSOC, LGSOC, EC, OCCC, and MOC) [46]. All this pointed out an inevitable use of comprehensive omics techniques for proper classification of cell lines as models for tumor types which they represent. For instance, a genome-wide analysis of 45 OC cell lines, including some rare subtypes, revealed that these cell lines were largely representative of primary ovarian cancers. Analysis of mutation signatures demonstrated that serous, mucinous, and undifferentiated tumor subtypes showed the age-related signature, while clear cell and serous OCs also had a mismatch repair-associated signature. The most frequently mutated gene is *TP53*, followed by *ARID1A*, *PIK3CA*, *SMAD4*, *KRAS*, *APC*, *CREBBP,* and *PPP2R1A*. Frequent deletions include *CDKN2A*, *CDKN2B*, *ERBB4*, *NF1*, *NF2*, *CDC73*, *EZH2* and *STK11* [47]. This is somewhat consistent with the data available from The Cancer Genome Atlas (TCGA) project, which encompasses 489 HGSOC samples. The most frequently mutated gene in this dataset is also *TP53*, followed by *NF1*, *BRCA1*, *BRCA2*, *RB1,* and *CDK12* [48]. TCGA analysis also revealed that somatic copy number abnormalities are common in HGSOC, resulting in frequent amplification of *CCNE1*, *MYC,* and *MECOM* genes, and deletions of *PTEN*, *RB1,* and *NF1* [48]. Methylation analysis revealed increased promoter hypermethylation of *AMT*, *CCL21*, *SPARCL1*, *RAB25,* and *BRCA1* [48]. Considering the limitations of the TCGA subset, an omics-wide integrated analysis was performed on 96 primary invasive early-stage OCs divided into OCCC, EC, HGSOC, LGSOC, and MOC. Unique DNA methylation patterns were identified for each subtype, with OCCC showing the highest methylation, and HGSOC the lowest. MOC showed the highest average number of copy number alterations. RNA expression was able to classify the different histotypes, but the differences were more evident when using the DNA methylation heatmap [49].

One of the best and largest resources on cell lines is the Cellosaurus (https://web.expasy.org/cellosaurus/) (accessed on 7 October 2021) [50]. Furthermore, the Cancer Cell Line Encyclopedia (https://sites.broadinstitute.org/ccle/) (accessed on 7 October 2021) [51] and the COSMIC Cell Line Project (https://cancer.sanger.ac.uk/cell_lines) (accessed on 7 October 2021) [52] contain many different omics information obtained from cell lines. However, not all OC cell lines in those two databases are fully classified, so a combined survey of different databases and original publications is needed to discover to which OC subtype a cell line of interest belongs.

## 3. Long Non-Coding RNAs: Classification and Mechanisms of Action

Non-coding RNAs (ncRNAs) are a large class of RNA molecules that are translated but do not encode proteins. This class includes a wide range of different RNA molecules, which can be broadly classified into infrastructural ncRNAs and regulatory ncRNAs. Infrastructural ncRNAs include transfer RNAs (tRNAs), ribosomal RNAs (rRNAs), and small nuclear RNAs (snRNAs) and their functions are well-known and have been examined in detail [53]. Regulatory ncRNAs include molecules such as small nucleolar RNAs (snoRNAs), microRNAs (miRNAs), small interfering RNAs (siRNAs), P-element-induced wimpy testis interacting (PIWI) RNAs (piRNAs), and long non-coding RNAs (lncRNAs). SnoRNAs, miRNAs, and piRNAs are collectively named small non-coding RNAs (sncRNAs).

LncRNA molecules are transcribed by RNA Polymerase II and processed in a way that resembles mRNA processing, as they are often 5′ capped, 3′ polyadenylated, and spliced similarly to mRNAs [54]. It has been demonstrated that 98% of lncRNAs are spliced [55], while only 40% of lncRNA transcripts are non-polyadenylated [56,57]. Furthermore, lncRNAs show a bias toward two-exon transcripts, with slightly longer exons and longer introns than protein-coding genes which are often alternatively spliced. Their level of expression is generally lower than that of protein-coding genes and is more tissue-specific [55,58,59].

**Figure 1:**
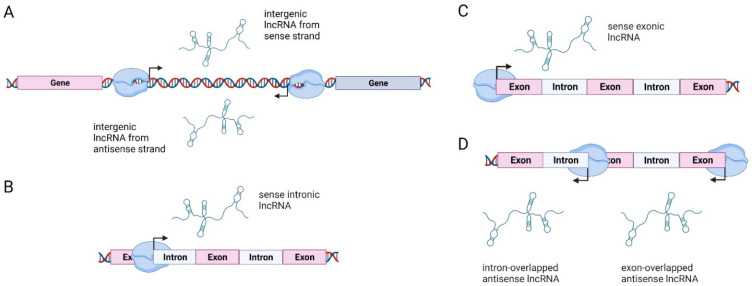
Categories of lncRNAs based on their location in respect to protein-coding genes. (**A**) Long intergenic non-coding RNAs (lincRNA) are transcribed intergenically from both strands. (**B**) Sense intronic transcripts are located within introns of coding genes without intersecting with exons. (**C**) Sense overlapping transcripts overlap with the exons of coding genes on the same strand. (**D**) Antisense RNAs are located within the exons and introns of protein-coding genes but on the opposite strand. Created with BioRender.com.

Biogenesis of lncRNAs is based on their location in respect to protein-coding genes, and lncRNAs can thus be classified into four categories: (1) long intergenic non-coding RNAs (lincRNAs), transcribed intergenically from both strands; (2) sense intronic transcripts, located within introns of coding genes without intersecting with exons; (3) sense overlapping transcripts, which overlap with the exons of coding genes on the same strand; and (4) antisense RNAs, which are located within the exons and introns of protein-coding genes but on the opposite strand (so-called natural antisense transcripts or NATs) (Figure 1) [60,61,62,63]. The majority of lncRNA loci are located in intergenic regions [55]. A detailed explanation of lncRNA biogenesis can be found in [64].

Mutations and genomic rearrangements can affect the expression of lncRNA in the same way as they affect the protein-coding genes and can contribute to tumorigenesis. Aznaourova et al., have summarized a list of lncRNAs involved in various human diseases, including gynecological cancers [65]. Regulation of tumor progression by lncRNA can occur through modification of several mechanisms: the epigenetic regulation of genes (chromatin remodeling, histone modification, DNA methylation), the transcriptional regulation of genes (cis-regulation of nearby genes or trans-regulation of distant genes), and post-transcriptional regulation of gene expression (alternative splicing of pre-mRNA, stabilization of mRNA, and translation and stabilization of proteins) [7].

**Figure 2:**
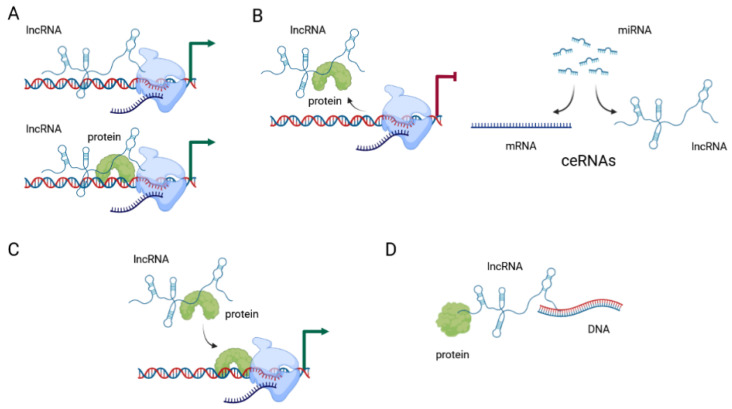
Functional roles of lncRNAs. (**A**) As signal molecules, lncRNAs act either alone or in combination with proteins like transcription factors to mediate the transcription of downstream genes; (**B**) As decoys, lncRNAs either bind to functional proteins to block their activity or compete with mRNAs for binding to miRNAs to block their inhibitory effects on mRNAs; (**C**) As guides, lncRNAs carry and locate functional proteins in the target area to perform their functions; (**D**) As scaffolds, lncRNAs guide different types of macromolecules to assemble complexes and facilitate their interactions. Created with BioRender.com.

Initial systematic large screening of lncRNA genes, using genome-wide chromatin-state maps by immunoprecipitation, identified over a thousand highly conserved lncRNAs [66]. The current estimate of the number of genes encoding lncRNA according to the GENCODE and FANTOM projects is between 17,957 and 27,919 genes [67]. Their functions are classified as either (1) RNA-based, where lncRNA interacts with DNA, RNA, or proteins, (2) gene-regulatory, where lncRNA modifies the activity of regulatory elements, or (3) transcription-based, where the process of transcription influences gene activity [67]. They can function as signals, decoys, guides, or scaffolds (Figure 2) [7].

LncRNAs are considered to signal molecules when they regulate the transcription of target genes. In this context, lncRNA can exert this effect alone, or in combination with transcription factors. Their expression is triggered by specific conditions, and they in turn trigger specific responses to those conditions. The best-known example of this type of regulation is the lncRNA *XIST*, which is involved in X-chromosome inactivation by direct interaction with the chromatin [68]. Other examples include lncRNA *HOTAIR*, which is transcribed from the homeobox transcription factor cluster (HOX) in tightly controlled conditions; *PANDAR*, which is induced by DNA damage; *LINC-ROR*, which is associated with reprogramming to induced pluripotent stem cells; and *COLDAIR*, which is induced by cold [69].

When acting as decoys, they usually block specific molecular pathways by binding to a specific protein or RNA and impairing its function. This is the most common type of regulation that lncRNAs exert on their targets. The targets are most often miRNA molecules, and lncRNAs antagonize the miRNA they target by sequestering the miRNA away from their intended mRNA targets [69]. Competing endogenous RNAs (ceRNAs) or “molecular sponges” act as competitive inhibitors which block the activity of the target mRNAs. Most often this mechanism acts to “sponge” various miRNA molecules, thereby removing them from their intended targets and suppressing their effect [70].

Guide lncRNAs direct the localization of ribonucleoprotein complexes to specific targets. This effect can be achieved in cis, when they affect neighboring genes, or in trans when the effect is on distant genes. These effects are mediated through changes in the structure of chromatin. Regulation in cis involves specific interaction between the lncRNA and chromatin at its promoter, leading to accumulation of proteins involved in histone 3 lysine 9 (H3K9) methylation and gene silencing. Regulation in trans often targets proteins of the Polycomb Repressive Complex (PRC) and changes their localization, activity, and occupancy, leading to changes in chromatin structure in trans. *HOTAIR* is one such lncRNA, often found overexpressed in tumors and associated with cancer metastases [69].

When acting as scaffolds, they facilitate the interaction of different molecules and proteins. *CDKN2B-AS1*, a lncRNA located in the *INK4b/ARF/INK4a* locus, recruits multiple chromatin-modifying proteins and thereby modulates transcriptional activity of the locus [69]. It is important to emphasize that lncRNAs employ more than one mechanism of action to exert their effects, so one function does not necessarily exclude all others. There are potentially other yet undescribed mechanisms by which lncRNAs can affect gene and protein expression and function.

Even though lncRNAs were originally defined as RNA which do not encode proteins, recent reports suggest that some lncRNAs contain open reading frames (ORF) and may encode peptides [71]. These peptides are involved in various signaling processes, e.g., mTOR signaling, regulation of Ca^2+^-ATPase, mRNA decay, and mitochondrial activity. They can also be involved in metabolic reprogramming of several cancers, such as colon, liver, esophageal, and breast cancer, and melanoma [71]. Furthermore, lncRNAs can also encode miRNAs, as exemplified by miR-675 which is transcribed from exon 1 of *H19* [72].

## 4. Intracellular and Extracellular Compartmentalization of lncRNAs

LncRNAs can be found in different cellular compartments, from the nucleus to cytoplasm and mitochondria, and even exported from the cell via exosomes. About 30% of lncRNAs are located in the nucleus, where they can interact with transcription factors, chromatin complexes, and heterogenous ribonucleoprotein complexes [73]. Within the cytoplasmic compartment, lncRNAs can be found in many structures and organelles. LncRNAs in ribosomes are most often associated with nonproductive initiation of translation, but small peptides may be produced from open reading frames in individual lncRNA. Additionally, lncRNAs in the ribosome may act as regulators of translation [74]. So far, several lncRNAs that are transcribed from the mitochondrial genome, and about 20 nuclear-encoded lncRNAs that affect mitochondrial biology have been described [75]. These include some ubiquitous lncRNAs that are involved in many oncogenic processes, such as *SAMMSON*, *HOTAIR*, *H19*, *HOTTIP*, and *MEG3*, but also some specific ones, like *CEROX1* (cytoplasmic endogenous regulator of oxidative phosphorylation 1), which affects the process of oxidative phosphorylation. Mitochondria-encoded lncRNAs are either antisense mitochondrial transcripts, like *lncND5*, *lncND6*, *lncCYB*, and *MDL1AS*; chimeric mitochondrial transcripts, like *ASncmtRNA-1* and *ASncmtRNA-2*; or putative mitochondrial DNA-encoded lncRNAs, like *LIPCAR* **[75]**.

Exosomes are bi-layered membrane vesicles secreted from the cells, 30–150 nm in diameter, which can contain a wide range of molecules usually found within the cells. They are generated by the budding of the endosomal membrane. LncRNAs are packed inside exosomes whereby they interact with RNA-binding proteins through specific motifs. Exosomes are used in cell-to-cell communication, and whatever is packed inside an exosome may be delivered to a receiving cell and trigger a specific molecular response in that cell. In the case of tumor-derived exosomes, these responses range from tumor growth, invasion, and metastasis to angiogenesis and reprogramming of the tumor microenvironment. Furthermore, several lncRNAs (*UCA1*, *FAM225A*, *RAMP2-AS1*, *POU3F3*, *HOTAIR*, *CCAT2*) have been identified that affect the endothelial cells of blood vessels and stimulate angiogenesis [76]. The cancer-associated fibroblasts-derived exosomes were shown to carry lncRNA *H19*, which is highly expressed in tumor stroma in comparison to tumor tissue. This lncRNA promotes stemness and chemoresistance of cancer cells and increases the frequency of tumor-initiating cells [77].

## 5. The Role of lncRNAs in Various Biological Processes Related to Ovarian Carcinogenesis

Each hallmark of cancer can be modulated by lncRNA activity, thus leading to increased proliferation, viability, growth suppression, motility, immortality, and angiogenesis [78]. It has been debated whether lncRNAs can act as initiators of tumorigenesis, or if they are dysregulated as a consequence of tumorigenesis. The Cancer LncRNA Census contains a compilation of 122 GENCODE lncRNAs associated with the initiation of tumorigenesis, some (63.1%) acting as oncogenes, some (28.7%) as tumor suppressors, and some (8.2%) with evidence of both activities depending on tumor type. LncRNAs *HOTAIR*, *MALAT1*, *MEG3*, and *H19* are associated with a large number of cancer types [79]. The same study identified tumor-causing mutations in several lncRNAs, supporting their role in tumor initiation [79]. A recent literature survey of Salamini-Montemurri et al., has identified 215 lncRNAs being involved in OCs, of which for 157 there is experimental proof [8]. Apart from initiation, lncRNAs have been associated with many biological processes associated with metastasis [80], such as the immune response [73], radiation response [81], epithelial to mesenchymal transition (EMT) and stemness [82], cell-to-cell communication, and regulation of the microenvironment in which cells reside [77].

It is generally said that in the context of OC, there is proof that lncRNAs are involved in all the hallmarks of cancer except ‘genomic stability and mutation’ and ‘enabling replicative immortality’ [8]. Several lncRNAs showed influence on the size and weight of ovarian tumors in vivo, such as *AB073614* [83], *EPB41L4A-AS2* [84], *GAS5* [85], *LINC00565* [86], *TINCR* [87] and *TPT1-AS1* [88]. Loss of cell cycle control, related to proliferative signal maintenance and evasion of growth suppressors, is in OC associated with lncRNAs *MNX1-AS1* [89], *SPRY4-IT* [90], *KB-1471A8.2* [91], and *CASC15* [92]. Avoidance of cell death which causes cell immortalization has mostly been studied in the context of apoptosis. It was shown that lncRNA *LNCRNA-ATB* [93] can regulate apoptosis in OC. Also, *GAS5* can trigger a highly inflammatory type of programmed cell death called pyroptosis [94], while some lncRNAs can either induce (*MEG3* [95] and *MALAT1* [96]) or inhibit (*HOTAIR* [97], *HULC* [98] and *RP11-135L22.1* [99]) autophagy in OC. Some lncRNAs can control cancer cell metabolism by regulating key enzymes in metabolic pathways, mainly stimulating glycolysis (the Warburg effect) [100]. For instance, it was shown in OC that lncRNAs *LINC00504* [101], *NRCP* [102], *LINC00092* [103], and *H19* [104] can activate different enzymes in the glycolysis pathway from glucose to pyruvate. It has been shown that *MALAT1* promotes angiogenesis in OC by inducing the expression of *VEGF* and *FGF* [105], while *DANCR* [106] and *HNF1A-AS1* [107] can promote it by inducing *VEGF* and *SEMA4D* expression.

LncRNAs play a role in the regulation of immune response, where they are involved in several mechanisms of action. A major role is antisense silencing associated with *DICER* and molecular sponge function for reducing the regulatory effects of miRNAs [73]. Many stages of the immune response can be targeted and regulated by lncRNAs. Tumor antigen release, more specifically, the expression of chaperone calreticulin, can be inhibited by lncRNA expressed from the *RB1* promoter (*RB1-DT*). Antigen presentation can be affected by the specific reduction of the abundance of antigen-presenting cells (lincRNA *LINC01139*) or by the regulation of pro-inflammatory cytokines. *EGILA* can affect the differentiation of T lymphocytes, thereby affecting immune cell priming and activation. LncRNAs can also affect immune cell migration and infiltration, and affect recognition and attack of cancer cells [108]. *HOTTIP* was cited as the only lncRNA associated with the avoidance of immune surveillance by increasing *IL6* expression in ovarian tumor cells, which triggers *PD-L1* expression through the STAT3 pathway in neutrophils [109]. Therefore, targeting specific lncRNAs or modifying their activity to boost the immune response or increase recognition of tumor cells may be a potential therapeutic approach in ROCs.

Resistance to radiation can be modulated positively and negatively by the lncRNAs. Some lncRNAs, such as *GAS5*, increase the radiosensitivity of cancer cells through their sponging mechanisms. In contrast, other lncRNAs, such as *HOTAIR* or *TUG1*, can increase radioresistance by sponging specific miRNAs. Expression of lncRNAs can also be used as biomarkers of radiation damage, as lncRNA *TP53COR1*, *TUG1* and *MEG3* have been found to be upregulated after radiation damage by multiple studies [81]. Increasing the radiation sensitivity of tumor cells or increasing radiation resistance in healthy tissues by targeting specific lncRNA targets could be a promising approach for the treatment of various cancers, including ROCs.

EMT is a reversible process during which epithelial cells acquire mesenchymal characteristics, including reduction of cell-cell contacts, changes in cell morphology, and increase of migratory capability. This transition is considered one of the crucial steps during tumor invasion and metastasis. The process is reversible, and under the right conditions, the cells can transition back from mesenchymal to the epithelial phenotype, resulting in mesenchymal to epithelial transition (MET). EMT process is regulated by specific transcription factors such as SNAIL, SLUG, TWIST1, TWIST2, ZEB1, and ZEB2 [110]. Cancer stem cells (CSC) are a subpopulation of tumor cells with the capacity for self-renewal, asymmetric division, and multipotency. This stemness phenotype is often acquired through the EMT process and regulated by transcription factors SOX2, OCT4, NANOG, KLF4, and LIN-28. Many of these EMT and CSC regulators can be controlled by lncRNA such as *HOTAIR*, *H19*, *MALAT1*, *LINC-ROR*, *HOTTIP*, *NEAT1*, *LNCRNA-ATB*, and others [82].

Cell-to-cell communication and the microenvironment are important factors during tumor progression and metastasis. Tumor cells interact with their environment and various cell types in their surroundings. The most abundant and relevant cell types in this microenvironment are the cancer-associated fibroblasts (CAF), which respond to the cytokines produced by the tumor to support tumor growth and dissemination. Many lncRNAs are upregulated in the CAF compared to normal fibroblasts, such as *MALAT1*, *NEAT1*, *H19*, *GAS5*, and *MEG3* [77,111]. The communication also goes in the opposite direction, from tumor cells to fibroblasts, reprogramming them into CAF-like cells [77]. Interrupting this cell-to-cell communication between tumor and stromal cells through lncRNAs is also a promising approach to tumor treatment, as the microenvironment plays a major role in the ability of tumors to grow, progress, and ultimately metastasize.

Post-translational modifications (PTMs) of proteins are involved in several biological phenomena including signaling processes that occur at different stages of tumor development [112,113]. Although the mechanism of action is not fully discovered, change in the PTMs affects protein activity, stability, protein-protein, and protein-nucleic acid interactions.

LncRNAs interfere with PTMs in the cytoplasm [114]. They may downregulate the PTMs by interacting with enzymes involved in PTMs and/or blocking the modification sites. The size of PTMs and associated proteoforms in biological systems are too large and there is no model system that shows the dynamic interactions in diseases. However, IncRNA-interacting genes obtained from bioinformatical analyses can be used to assess the potential IncRNA-PTMs relationship in OC.

Histones are subjected to several PTMs including methylation, acetylation, phosphorylation, and ubiquitination [115]. It has been previously shown that methyl-CpG-binding domain protein 1 (MBD1) interacts with *H19* through repressing the methylated regions. It also affects the adjacent insulin-like growth factor 2 gene and associated proteins [114]. Both alterations in MBD1 [116] and insulin-like growth factors are linked to OC. Histone acetylation is another key chromatin modification causing epigenetic variation that is implicated in OC pathogenesis [117,118]. LncRNAs can change this process either by interrupting the histone acetylation process, such as *LNCPRESS1* binding to SIRT6 and preventing its binding to histones [119], or by acting as scaffolds regulating histone acetylation and methylation, such as *GClnc1* which acts as a scaffold for WDR5, and KAT2A proteins [120].

It is well-known that phosphorylation of proteins is one of the most common mechanisms that regulate protein activity, and phosphorylation is often dysregulated in cancer. AKT and mTOR phosphorylation have been detected as potential biomarkers for OC and tumor growth [121]. *LncRNA-AKT* interactions were experimentally shown in lung cancer [122] which might be a sign for similar connections for other cancers including ovarian.

The majority of the proteins are glycosylated and glycosylation plays a crucial role in cancer biology [122] and serves both as a diagnostic and prognostic biomarker for OC [122,123,124,125]. Protein-specific glycosylation studies have discovered changes in Immunoglobulin G (IgG), haptoglobin beta-chain, alpha1-acid glycoprotein, and alpha1-antichymotrypsin glycosylation in OC [126]. Although the role of IncRNAs has not been completely explored in OC, a link between lincRNAs and N-Glycosylation of IgG has been detected [127].

Both bioinformatical and experimental studies have discovered that lncRNAs prompt PTMs at different levels. The outcomes need to be further investigated to unravel the possible mechanisms leading to accurate diagnosis and effective treatments.

LncRNAs, primarily through their crosstalk with miRNAs, regulate the osteogenic differentiation of mesenchymal stem cells (MSCs), a population of stromal cells in the bone marrow, and other anatomical regions such as adipose tissue and apical papilla of the tooth [128]. MSCs are capable of self-renewal and differentiation into multiple cell lineages, of which osteoblasts are important for bone development, homeostasis, and regeneration [129]. Their differentiation is mediated by several signaling pathways of which TGF-β/BMP and WNT/β-catenin pathways are central [130]. Interestingly, several lncRNAs which were found to be differentially expressed in OCs, such as *H19*, *NEAT1*, *MALAT1*, *HOTAIR*, or *XIST*, participate in this cellular process [131]. Although osteogenic differentiation is primarily associated with the development of osteosarcoma [132], MSCs could be recruited to the tumor microenvironment and can both restrict [133] and promote solid tumor growth [134]. In OCs, carcinoma-associated MSCs have been identified in ovarian tumor tissue samples and it was proven that they can regulate ovarian cancer stem cells proliferation and tumorigenesis through altered production of BMP [135]. It was also shown that umbilical cord MSCs inhibited the growth of EC cell line TOV-112D [136], while bone marrow MSCs reduced the growth rate of cisplatin-resistant EC SKOV3 cells [137]. Bu et al. showed that also endometrial MSCs derived from human menstrual blood can attenuate tumor growth of EC cell line SKOV3 and postulated that observed intrinsic anti-tumor properties of adult MSCs could be utilized for developing an MSC-based therapy for treating OCs [138].

## 6. LncRNAs Associated with Common and Rare Ovarian Cancers

TCGA data has been used to analyze lncRNA in different tumor types, but OC has not been analyzed in more detail because the TCGA database lacks the appropriate healthy tissue controls for HGSOC samples, thus making the differential expression analysis impossible for OC [139,140]. However, somatic copy number alterations are frequent in OC, and lncRNA *BCAL8* has been found amplified in breast cancer and associated with poor clinical outcomes of OC [139]. HGSOC can be divided into four distinct subtypes: immunoreactive, characterized by *CXCL11*, *CXCL10,* and *CXCR3* expression; differentiated, characterized by *MUC16* and *MUC1* expression; proliferative, characterized by *HMGA2*, *SOX11*, *MCM2,* and *PCNA* overexpression and *MUC1* and *MUC16* downregulation; and mesenchymal, characterized by high expression of *HOX* genes, *FAP*, *ANGPTL2* and *ANGPTL11* [48]. Mesenchymal subtypes often show upregulation of *MIAT* (also known as *gomafu* in humans), proliferative types show downregulation of *NEAT1* and *UCA1*, while serous subtypes show frequent amplification of *OVAL* [140].

Several lncRNAs have been found to have an effect in a wide range of human cancers, including many gynecological cancers. In OC, a specific signature of six lncRNA has been identified by a bioinformatical analysis. This signature associates *RUNX1-IT1*, *MALAT1*, *H19*, *HOTAIRM1*, *LOC100190986,* and *AL132709.8* lncRNAs [141]. Of these, *MALAT1* and *H19* have been extensively studied in OC, along with several other lncRNAs: *HOTAIR*, *NEAT1*, *XIST*, *MEG3,* and *UCA1*. Most extensively analyzed lncRNAs in OC have been listed in Table 2, and the lncRNAs more specific for ROCs are summarized in Table 3.

### 6.1. H19

A locus for the *H19* imprinted maternally expressed transcript produces a 2.3 kb long RNA transcript, which is abundantly expressed during embryonic development and downregulated after birth. It is a paternally imprinted gene, but imprinting loss, and subsequent overexpression, have been associated with ovarian tumors [142,143]. Knockdown of this lncRNA promotes G2/M cell cycle arrest, induces apoptosis of OC cells, and inhibits cell growth [143]. *H19* can act as a competing endogenous RNA of miR-370-3p, resulting in the promotion of TGFB1-induced EMT [144], and as an inhibitor of a tumor-suppressor miRNA let-7, resulting in increased tumor cell migration and invasion [145]. H19 is elevated in OC cells resistant to cisplatin [146]. Its downregulation leads to increased sensitivity to cisplatin [147,148]. Polymorphisms in the *H19* locus are associated with platinum-based chemotherapeutic response [149]. *H19* can also affect cancer cell metabolism, through the sponging effect of miR-324-5p, which regulates PKM2, a major contributor to the Warburg effect [104]. Although most of these studies were performed on serous epithelial ovarian cancer cell lines, the role of *H19* has also been examined in some ROCs. *H19*-overexpressing choriocarcinoma cells were found to be more tumorigenic in vivo, even though there was no difference in their clonogenicity in the in vitro assays. There is a selection of cells expressing high levels of *H19* during the microevolution of tumor progression, which suggests that this lncRNA does not act as a tumor-suppressor [150]. Loss of *H19* imprinting has also been demonstrated in pediatric germ cell tumors, and it may reflect the origin of these tumors in different stages of germ cell development [151,152,153]. Benign ovarian teratomas show a varying degree of *H19* hypomethylation, and DNA prepared from cultured teratoma cells shows extreme hypomethylation of the *H19* locus [154]. Ovarian granulosa cell tumor cell line KGN has been used to demonstrate that lncRNA *H19* binds to miR-19b, resulting in upregulation of CTFG, increase in cell proliferation, d reduction in the rate of apoptosis in these cells [155].

### 6.2. HOTAIR

HOX transcript antisense RNA (*HOTAIR*) has shown some potential as a diagnostic and predictive biomarker. This lncRNA acts as a molecular scaffold and binds the Polycomb Repressive Complex 2 (PRC2) on its 5′ domain and the lysine-specific histone demethylase 1A (LSD1)/CoREST/REST complex on its 3′ domain, bringing them in close proximity and methylating lysine 27 and demethylating lysine 4 of histone H3, leading to consequent gene silencing [156,157]. Expression of *HOTAIR* promotes proliferation, stemness, and epithelial-to-mesenchymal transition of OC cells [158,159,160]. The transcription of *HOTAIR* is induced by estrogen, making it a relevant lncRNA in the context of gynecological cancers [161]. It has been associated with poor prognosis and tumor metastasis in epithelial ovarian cancer and cervical cancer [162,163,164]. Its prognostic value has been evaluated by a meta-analysis in four estrogen-dependent tumor types (breast, ovarian, cervical, and endometrial), and it may be a predictor of poor prognosis [165]. Specific genetic variants of *HOTAIR* may in some populations affect OC susceptibility [166,167]. *HOTAIR* expression is also associated with chemoresistance in clinical samples and in vitro models, and its knockdown can lead to increased sensitivity to cisplatin and carboplatin both in vitro and on a mouse xenograft model [97,159,168,169]. A peptide nucleic acid (PNA) designed to specifically block the activity of *HOTAIR* has been successful in reducing ovarian tumor growth in vitro and in vivo, and improved survival of xenograft mice, and has been suggested as a potential new therapeutic approach for the treatment of OC [170]. The potential use of this lncRNA as a biomarker has led to the development of various methods for detection of this lncRNA from samples derived from patients with OC, primarily plasma [171,172].

### 6.3. MALAT1/NEAT2

Metastasis-associated lung adenocarcinoma transcript 1 (*MALAT1*), also known as nuclear-enriched abundant transcript 2 (*NEAT2*), is another lncRNA significantly overexpressed in various cancers [173,174], and has been extensively studied in OC. A meta-analysis revealed that *MALAT1* could be a novel biomarker in various cancers, including ovarian [175]. It can be detected in the plasma, and it has been suggested as a potentially useful marker for OC metastases [176]. Metastatic EOC cells show increased expression of *MALAT1* and increased secretion through their exosomes, and this activates angiogenesis-related genes in the endothelial cells [105]. *MALAT1* is highly overexpressed in OC and is associated with the FIGO stage. Its overexpression leads to increased cell proliferation, migration, and invasion [177,178]. It can act through sponging several different miRNA molecules, all resulting in increased proliferation and survival of cancer cells: miR-22 [179], miR-506 [180], miR-200c [181], miR-143-3p [182], miR-200a [96], miR-211 [183], miR-503-5p [184]. *MALAT1* has also been found to downregulate RBFOX2, which leads to the alternative splicing of the KIF1B and production of the pro-apoptotic long isoform of KIF1B, ultimately resulting in inhibition of anoikis [185].

Other signaling pathways which are directly or indirectly affected by *MALAT1* include the PI3K-AKT pathway [186] and the ERK/MAPK pathway [187]. *MALAT1* has also been implicated in drug resistance of OC, as it was found upregulated in cisplatin-resistant OC cells, where it sponges miR-1271-5p, leading to upregulation of E2F5 and increased proliferation, migration, and invasion [188]. *MALAT1* is also upregulated in OC spheroids when compared to their adherent counterparts, suggesting a role of *MALAT1* in cancer cell stemness [189]. Knockdown of *MALAT1* can restore chemosensitivity of OC to cisplatin through the inhibition of the NOTCH1 signaling pathway [190]. Apart from the effects of *MALAT1* investigated in OC in general, some ROCs have also been investigated. Experiments on the KGN cell line demonstrated that *MALAT1* is upregulated in these cells and involved in the maintenance of proliferation and viability as well as inhibition of autophagy [191]. *MALAT1* knockdown induces upregulation of p21, p53, p-JNK, and p-ERK1/2, and downregulation of CDK2, cyclin D1, and p-P38 MAPK protein levels [187]. In choriocarcinoma *MALAT1* binds to miR-218, leading to upregulation of FBXV8 oncogene and cell proliferation [192]. In KGN cells knockdown of *MALAT1* results in inhibition of the ERK/MAPK pathway leading to the inhibition of cell proliferation and cell cycle progression [187].

### 6.4. MEG3

Maternally expressed 3 (*MEG3*) lncRNA has been reported as a tumor suppressor. Its major role is in the positive regulation of the tumor suppressor gene *TP53* [193]. *MEG3* is downregulated in many cancers, such as breast [194], cervical [195], gastric [196], lung [197], and EOC [198]. Downregulation in EOC is achieved through hypermethylation of the *MEG3* promoter, as treatment with demethylating agent 5-aza-2-deoxycytidine increases *MEG3* expression. Increased expression of *MEG3* suppresses proliferation and growth and induces apoptosis, and suppresses tumorigenesis in vivo [95,198]. Upregulation of *MEG3* induces expression of *PTEN* and *LAMA4*, leading to inhibition of cell proliferation, cell cycle arrest, and induction of apoptosis [199,200]. On the other hand, high expression of *MEG3* is associated with better progression-free survival and overall survival in HGSOC, even though the same authors demonstrated that MEG3 is downregulated in HGSOC cell lines compared to the normal fallopian tube and ovarian cell lines, and upregulation of MEG3 in HGSOC cells leads to inhibition of tumor growth in vitro and in vivo [201]. It can bind directly to ATG3 mRNA and protect it from actinomycin D-induced degradation [95]. *MEG3* is one of the three signature lncRNA that can be used to predict cisplatin resistance in OC. Upregulated *PVT1* and downregulated *TUG1* and *MEG3* have high sensitivity and specificity in predicting chemoresistance and are negatively associated with OS and progression-free survival [202]. Sensitivity to cisplatin can be restored with curcumin treatment, as it leads to demethylation of the *MEG3* promoter, sponging of miR-214 by *MEG3,* and consequent reduction of drug resistance [203]. *MEG3* expression can be negatively regulated by another lncRNA: *AGAP2-AS1*, which is upregulated in OC and participates in cancer cell proliferation [204].

### 6.5. NEAT1

Nuclear Paraspeckle Assembly Transcript 1 (*NEAT1*) is another lncRNA that is overexpressed in various cancers, including OC. *NEAT1* is upregulated in OC, positively correlated with FIGO stage, tumor grade, and distant metastasis. *NEAT1* expression level is an independent factor in predicting the overall survival of OC patients [205]. A meta-analysis of 1354 patients from 11 studies revealed that *NEAT1* expression is indeed significantly associated with poor overall survival, larger tumor size, lymph node metastasis, distant metastasis, TNM-stage, poor differentiation, and invasion depth [206]. Based on all this, *NEAT1* has been proposed as a prognostic biomarker in breast, ovarian, cervical, endometrial, and vulvar cancers [207]. It interacts with several miRNAs: miR-34a-5p [208], miR-124-3p [209], miR-382-3p [210], miR-506 [211], miR-1321 [212], miR-4500 [213] and miR-365 [214]. *NEAT1* is also involved in the development of resistance. In the platinum-resistant OC cells, *NEAT1* binds miR-770-5p, leading to upregulation of PARP1. Knockdown of *NEAT1* expression results in a reduction in xenograft tumor growth as well as increases sensitivity to cisplatin [215]. In the case of paclitaxel (PTX) resistance, *NEAT1* is upregulated in PTX-resistant OC tissues and cells and binds to miR-194. *NEAT1* knockdown enhanced the sensitivity of cells to PTX through the miR-194/ZEB1 axis [216]. The potential of *NEAT1* as a possible diagnostic marker has been proposed in several cancers, such as prostate [217] and breast [218]. For EOC, a serum biomarker panel that combines gene and protein expression was proposed as a method for early detection of EOC, and *NEAT1* is one of the proposed lncRNA in this panel [219].

### 6.6. UCA1

Urothelial cancer associated 1 (*UCA1*) is an oncogenic lncRNA found to be upregulated in many solid tumors. A meta-analysis revealed that upregulation of *UCA1* is negatively associated with overall survival and progression-free survival in many cancers, including OC [220]. *UCA1* acts as a sponge for miR-485-5p, which in turn upregulates the levels of MMP14, and this upregulation of MMP14 is an important factor in OC metastasis [221,222]. It can also bind directly to AMOT, a known regulator of YAP, and promote activation of YAP and subsequent transcription of target genes [223]. It has been suggested as a potential new biomarker and therapeutic target of OC, especially in the context of drug resistance where it may serve as an indicator of response to therapy [224,225]. For example, *UCA1* expression induces cisplatin resistance in OC cell lines. This could be mediated through the upregulation of SRPK1 [226] or the miR-143/FOSL2 axis [227]. Paclitaxel resistance has also been associated with *UCA1* expression, by regulating the miR-654-5p/SIK2 axis [228] and/or the miR-129/ABCB1 axis [229].

### 6.7. XIST

X inactive specific transcript (*XIST*) regulates X-chromosome inactivation, by acting as a scaffold for repressive epigenetic factors, and as many as 30 different RNA-binding proteins are predicted to bind with *XIST*. *XIST* RNA contains structured regions, or Xist motifs, which are crucial for its function. Of these, the A-repeat (RepA) folds into a stem-loop structure and is required for gene silencing [230]. A meta-analysis has demonstrated that *XIST* is associated with poor overall survival, larger tumor size, increased distant metastases, and advanced tumor stage in a range of cancers [231]. In OC, there are conflicting results. *XIST* has been found to be downregulated and correlated to better prognosis in OC in one study [232], while another study shows that *XIST* was upregulated in OC tissues and cell lines and is suggested as an independent predictor of prognosis for OC patients [233]. Studies regarding the role of *XIST* in OC are also contradictory–some claim upregulation of *XIST* leads to stimulation and increased proliferation of OC cells, while others claim it suppresses OC proliferation and tumor growth [233,234,235,236,237].

**Table 2:** The lncRNAs frequently involved in OCs and their mechanisms of action. OC type includes rare OC types (GCT–granulosa cell tumor) and common OC types (HGSOC–high-grade serous ovarian carcinoma, EOC–epithelial ovarian cancer, EC–endometrioid carcinoma), while the abbreviation OC refers to papers that did not define specific OC subtypes, and OCSC refers to ovarian cancer stem cells.

lncRNA	Target	OC Type	Mode of Action	Effect	Role	Reference
*H19*	miR-370-3p	EC, HGSOC	ceRNA	Promotes *TGFB1*-Induced EMT	Oncogene	[144]
	miR-324-5p	EC	ceRNA	Promotes Warburg Effect through *PKM2*	Oncogene	[104]
	miR-19b	GCT	ceRNA	Increased Expression of *CTGF*, Resulting in Cell Proliferation and Reduced Rate of Apoptosis	Oncogene	[155]
	let-7	EC, OCSC	ceRNA	Promotes Tumor Cell Migration and Invasion	Oncogene	[145]
*HOTAIR*	PRC2	/	Scaffold	The trimethylation of the H3K27 Histone and Consequent Gene Silencing	Oncogene	[156]
*MALAT1*	miR-22	EOC	ceRNA	Increased Cell Proliferation, Migration, Invasion, Tumor Growth, and Metastasis	Oncogene	[179]
	miR-506	OC	ceRNA	Upregulation of iASPP and Cell Proliferation	Oncogene	[180]
	miR-200c	EC, EOC	ceRNA	Increased Invasive Capacity	Oncogene	[181,238]
	miR-143-3p	EOC	ceRNA	Upregulation of *CMPK*	Oncogene	[182]
	miR-200a	EC	ceRNA	Promotes Autophagy and Invasion	Oncogene	[96]
	miR-211	EC, HGSOC	ceRNA	Upregulation of *PHF19*, Leading to OC Progression	Oncogene	[183]
	miR-503-5p	EOC	ceRNA	Promotes Proliferation and Inhibits Apoptosis Through the JAK2-STAT3 Pathway	Oncogene	[184]
	miR-1271-5p	HGSOC, EC	ceRNA	Upregulation of *E2F5* Expression, Mediates DPP-Resistant OC Development	Oncogene	[188]
	SRSF1	EOC	Scaffold	Downregulation of *RBFOX2*, Leading to Alternative Splicing of *KIF1B* Leading to Production of the Pro-Apoptotic Isoform	Oncogene	[185]
	YAP	EC	ceRNA	Inhibition of Nucleus-Cytoplasm Translocation, Resulting in Enhanced Activity and Promotion of Stemness Phenotype	Oncogene	[189]
	AMPK	GCT	Unknown	Proliferation, Viability, Inhibition of Autophagy, Downregulation of *AMPK*	Oncogene	[191]
	multiple	GCT	Signal Molecule	Downregulation of p21, p53, p-JNK, p-ERK1/2; Upregulation of CDK2, Cyclin D1, p-P38 MAPK	Oncogene	[187]
*MEG3*	ATG3	EOC	Scaffold	Protects *ATG3* mRNA from Degradation, Induces Autophagy	Tumor Suppressor	[95]
	miR-214	EOC	ceRNA	Reduction of Resistance to Cisplatin	Tumor Suppressor	[203]
	PTEN	EOC	Unknown	Upregulation of *PTEN*, Inhibition of Cell Proliferation, Induction of Apoptosis, Cell Cycle Block	Tumor Suppressor	[200]
	miR-219a-5p	OC	ceRNA	Downregulation of *EGRF*, Inhibition of Proliferation and Induction of Apoptosis	Tumor Suppressor	[239]
	miR-30e-3p	OC	ceRNA	Upregulation of *LAMA4*, Reduced Proliferation, Migration, and Invasion of OC Cells	Tumor Suppressor	[199]
	miR-205-5p	OC	ceRNA	Inhibition of Cell Viability, Migration, and Invasion, Induction of Apoptosis	Tumor Suppressor	[240]
*NEAT1*	miR-34a-5p	OC	ceRNA	Promotes Proliferation by Upregulating *BCL2*	Oncogene	[208]
	miR-124-3p	OC	ceRNA	Promotes Cell Proliferation and Invasion, *NEAT1* Expression is Stabilized by HuR Protein	Oncogene	[209]
	miR-382-3p	OC	ceRNA	Promotes *ROCK1*-Mediated Metastasis	Oncogene	[210]
	miR-506	EOC	ceRNA	Promotes Cell Proliferation and Migration, *NEAT1* is Stabilized by *LIN28B*	Oncogene	[211]
	miR-1321	OC	ceRNA	Increased Expression of *TJP3*, Enhances EMT, Invasion, and Migration	Oncogene	[212]
	miR-4500	OC	ceRNA	Increased Expression of *BZW1*, Enhances Cell Proliferation, Colony Formation, Migration, Invasion, and Glycolysis, Reduces Apoptosis	Oncogene	[213]
	miR-365	EC, HGSOC	ceRNA	Increased Expression of *FGF9*, Promotes Cell Proliferation and Angiogenesis	Oncogene	[214]
*UCA1*	miR-485-5p	EOC	ceRNA	Increased Expression of *MMP14*, Possible Role in Metastasis of OC	Oncogene	[221]
	AMOT	EOC	ceRNA	Enhances AMOT-YAP Interaction, Activation of YAP Target Genes,	Oncogene	[223]
	miR-143	OC	ceRNA	Upregulation of *FOSL2*, Increased Cisplatin Resistance	Oncogene	[227]
	miR-654-5p	OC	ceRNA	Upregulation of *SIK2*, Resistance to Paclitaxel	Oncogene	[228]
	miR-129	OC	ceRNA	Upregulation of *ABCB1*, Resistance to Paclitaxel	Oncogene	[229]
*XIST*	miR-149-3p	EOC	ceRNA	Upregulation of *FOXP3* Leading to OC Cell Proliferation	Oncogene	[235]
	miR-101-3p	HGSOC	ceRNA	Upregulation of C/EBPα and *KLF6* Leading to Macrophage Polarization to Affect Cell Proliferation of OC	Oncogene	[237]
	miR-214-3p	HGSOC	ceRNA	Suppression of Cell Proliferation, Invasion, Increased Chemosensitivity, Inhibition of Tumor Growth In Vivo	Tumor Suppressor	[236]
	miR-106a	OC	ceRNA	Decrease in Cell Proliferation and Activation of Apoptosis, In Vivo Tumor Growth Deceleration	Tumor Suppressor	[234]

### 6.8. Other lncRNAs Involved in Rare Ovarian Cancers

LncRNA growth arrest-specific 5 (*GAS5*) is upregulated in the plasma of patients with polycystic ovary syndrome (PCOS). This was also demonstrated in the ovarian granulosa cell tumor cell line KGN, where it leads to upregulation of IL6, and decreased apoptosis rate of these cells [241]. HLA complex P5 (*HCP5)* promotes cell proliferation and inhibits apoptosis in the KGN cell line through the miR-27a-3p-IGF1 axis [242]. In contrast, NPTN intronic transcript 1 (*NPTN-IT1*, or *lncRNA-LET*) is downregulated in KGN cells, and its overexpression inhibited cell viability and migration and promoted apoptosis [243].

Upregulation of long intergenic non-protein coding RNA 324 (*LINC00324*) has been found in immature ovarian teratocarcinoma (IOT) tissues and cells. *LINC00324* acts as a miR-214-5p sponge, thereby removing its inhibition of CDK6, CCND1, MDM2, and MDM4, consequently increasing IOT cell proliferation and decreasing apoptosis [244].

A study by Yan et al., identified neuroblastoma-associated transcript 1 (*NBAT1*) as a marker of favorable prognosis in OC. The study included 46 serous OC and 11 OC tumors of different origins, but there was no additional information on these other subtypes. However, the authors state that there was no difference between histological subtypes, suggesting that this marker is applicable for ROCs as well [245].

**Table 3:** Other lncRNAs with a role in ROCs. ROC analyzed in these papers include GCT–granulosa cell tumors, IOT-immature ovarian teratocarcinoma, and undefined (other).

lncRNA	Target	OC Type	Mode of Action	Effect	Role	Reference
*GAS5*	IL6	GCT	Unknown	Upregulation of IL6, Decreased Apoptosis	Oncogene	[241]
*HCP5*	miR-27a-3p	GCT	ceRNA	Proliferation, Inhibition of Apoptosis	Oncogene	[242]
*NPTN-IT1*	NF90	GCT	Scaffold	Reduced Cell Viability and Migration, Increased Apoptosis	Tumor Suppressor	[243]
*LINC00324*	miR-214-5p	IOT	ceRNA	Proliferation, Decreased Apoptosis	Oncogene	[244]
*NBAT1*	ERK1/2 and AKT Signaling Pathways	Serous and Other	Unknown	Inhibition of Cell Proliferation, Invasion, and Migration	Tumor Suppressor	[245]

## 7. Circulating lncRNAs as Diagnostic and Prognostic Biomarkers for OCs

Liquid biopsies are considered a minimally invasive means for managing cancer patients, and they can be applied in diagnosis, follow-up, and prediction to therapy. Liquid biopsies can be used for the detection of circulating tumor cells (CTCs), cell-free tumor DNA (ctDNA), and non-coding RNAs [246]. Circulating non-coding RNAs (miRNA and lncRNAs) have been proposed as potential biomarkers for the early detection of OC. In particular, multiple miRNA panels have been proposed as screening tools in clinical practice [247]. Several publications examine the possible role of lncRNAs in the diagnosis, prediction, and prognosis of OCs. Non-coding RNAs can be secreted from EOC cells via exosomes, and in turn exosomes from the serum of patients can be used for detection of these non-coding RNAs. Elevated exosomal levels of *MALAT1* in the serum of patients were highly correlated with advanced and metastatic subtypes of EOC, and an independent predictive factor for overall survival [105]. Exosomal *HIF1A-AS2* levels from the serum of EOC patients have been associated with poorer overall survival and suggested as a non-invasive predictive biomarker for unfavorable prognosis [248]. *LOXL1-AS1* was analyzed in the plasma of EOC patients, and its expression was associated with advanced FIGO stage, distant metastases, and short overall survival. It was proposed to be an independent diagnostic and prognostic factor in EOC [249]. A meta-analysis, which encompassed 1732 OC patients and 3958 controls, evaluated the diagnostic accuracy of ctDNA, miRNAs, and lncRNAs and found, albeit on a small number of studies, that lncRNAs were more accurate than miRNAs in diagnosing OC, with similar specificities. The authors claim that combining ctDNA, miRNA, and lncRNA biomarkers is the best option as it avoids the shortcomings of single biomarkers regarding sensitivity and specificity [250]. The major drawbacks and challenges in the detection of lncRNAs from liquid biopsies, and their use as biomarkers, is their low concentration in the serum/plasma and potential contamination with genomic DNA. Even though nowadays RNA extraction kits are able to remove the bulk of contaminating genomic DNA, nevertheless care must be taken during sample preparation, as contamination with genomic DNA in RNA preparations can lead to a false positive signal in qRT-PCR assays. This is especially true for lncRNAs encoded by a single exon, such as *MALAT1* [251]. Soda et al., have recently demonstrated that *HOTAIR* can be detected from plasma samples using electrochemical detection, and they propose that this assay could be used in a clinical setting for the detection of various lncRNA biomarkers [172]. It is also possible to detect microproteins encoded by lncRNAs in the extracellular vesicles in human plasma samples, and these microproteins could be used as diagnostic markers [252]. More studies of different circulating biomarkers in OCs are needed to assemble a good panel of miRNA/lncRNA targets for the diagnosis of OC and for determining their response to therapy. This could lead to better early detection and a personalized approach to therapy with a better outcome for OC patients.

Expression levels of circulating extracellular or exosome/EV lncRNAs associated with body fluids such as plasma/serum could serve as a potential biomarker for routine usage and a complementary liquid biopsy for the risk of ovarian tumor metastasis, recurrence, drug resistance, and potentially early detection. As single markers sometimes lack sensitivity and specificity, designing biomarker panels has been suggested as a good option for early detection, diagnosis, and prediction of recurrence. Such panels of lncRNAs have already been proposed for hepatocellular [253], liver [254], bladder [255,256], breast and cervical cancer [257]. A lncRNA panel as a candidate prognostic biomarker for OC has been proposed by Zhan et al. [258], but the focus was mainly on the construction of panels that can assess the response to therapy of OC patients, for example for platinum-based chemoresistance [259,260] or paclitaxel resistance [261]. LncRNA has the potential to be companion diagnostic tools alongside ctDNA and even CTCs in predicting recurrence and resistance, but how they could be incorporated into a screening program remains to be determined. In addition, a panel distinguishing between rare OC subtypes could prove to be most beneficial for diagnostic purposes.

## 8. Strategies for Targeting lncRNAs as a Treatment for OCs

The important role of lncRNAs in various biological processes related to carcinogenesis, together with their cancer-specific expression patterns, has made lncRNAs promising therapeutic targets. Therefore, many strategies have been developing for their targeting [262,263]. Generally, there are two main approaches, which have already been applied for targeting different lncRNAs in OC: to alter their expression level or to inhibit their interactions with other macromolecules [13].

Oncogenic lncRNAs are overexpressed in cancers so their expression can be suppressed using various, mostly nucleic acids-based techniques. Most commonly used methods are based on RNA interference (RNAi), like small interfering RNAs (siRNAs) or short/small hairpin RNAs (shRNAs) [264]. siRNAs are short, double-stranded (ds) RNAs that unwind into single strands (ss), bind to the RNA-induced silencing complex (RISC), and base-pair with targeting lncRNAs. That leads to Argonaute protein-dependent degradation of the target transcripts. Chemically synthesized siRNAs are usually directly delivered into the cytoplasm through transfection. shRNAs are produced as ss molecules 50–70 nucleotides long which form hairpin-like structures. They are usually encoded in DNA vectors and introduced into cells by plasmid transfection or viral transduction. They undergo processing and exert their mechanism of action similarly to siRNAs [265]. Presumably, all lncRNAs characterized in Table 2 and Table 3 as oncogenes, i.e., those which are over-expressed in OC and thus contribute to its initiation, tumorigenesis, or metastasis, could be targeted using RNAi methods. LncRNA silencing by siRNA or shRNA is probably the most used method for deducing lncRNA function in vitro [266].

LncRNA expression can also be suppressed using antisense oligonucleotides (ASOs), locked nucleic acid GapmeRs (LNA GapmeRs), or antagonists to natural antisense transcripts (antagoNATs) [267]. ASOs are ss antisense oligonucleotides made up of a DNA stretch at the central part with flanking RNA nucleotides. The DNA part with the target lncRNA, through Watson-Crick base pairing, forms a DNA/RNA heteroduplex which is cleaved by endogenous RNase H1 [268]. For instance, targeting lncRNA *MALAT1*, which is overexpressed in both common and rare OCs, by ASOs, inhibited tumor growth and metastasis of breast [269] and lung cancer [270]. LNA GapmeRs share structural and functional similarities with ASOs but have chemically modified LNA in flanking parts which increases their binding affinity and nuclease resistance [271]. LNAs have been constructed for targeting *XIST* [272], a lncRNA both up- and down-regulated in EOCs. NATs are coded from the opposite strand of the host gene locus and regulate expression of either sense transcripts of the same locus (cis-NATs) or transcripts from other genomic loci (trans-NATs). They mediate transcriptional silencing through histone-modifying complexes [273]. AntagoNATs are ss oligonucleotides designed to inhibit sense-antisense transcripts interactions and thus they can eliminate the epigenetic silencing effect of lncRNAs that act as NATs [274]. The first successfully applied antagoNAT was against *BDNF-AS*, a NAT that represses transcription of brain-derived neurotrophic factor (*BDNF*) gene [274]. This approach could potentially be used for regulating the expression of ovarian *BDNF*, which promotes survival, migration, and attachment of tumor precursors originated from *TP53*-mutated fallopian tube epithelial cells, precursors of HGSOC [275]. AntagoNATs could also be designed for targeting other NATs commonly dysregulated in common and rare OCs, such as *HOTAIR*, *MALAT1*, *MEG3,* and *GAS5* [273].

Other methods used for silencing lncRNA expression are mixmers and deoxy/ribozymes. Mixmers are built of chemically modified nucleotides like LNAs and different types of monomers. They sterically inhibit interactions between lncRNAs, ribonucleoproteins, or nucleic acids. They are used for preventing the formation of epigenetic remodeling complexes, altering gene expression and alternative splicing, repairing defective RNAs, and restoring protein production [276]. So far, the best-described example is a mixmer consisting of LNA interspersed with 2′-O-methyl nucleotides with a high-affinity for *SMN-AS1*, an antisense transcript that represses expression of survival motor neuron 2 (*SMN2*) gene by recruiting the PRC2 to its locus [277]. For treating OCs, a similar approach could be used, for instance, for targeting interactions between PRC2 and *HOTAIR* [156] or *XIST* [278]. Deoxyribozymes are enzymatic ssDNA molecules that bind target RNA through Watson-Crick base pairing and catalyze RNA cleavage (ribonucleases) [279]. Similarly, there exist engineered ribozymes that have better catalytic activities and more specific substrate recognition domains [280]. A site-specific deoxyribozymes have been designed to cleave RNAs that have *N*^6^-methyladenosine (m6A) modifications [281]. In the meantime, it was discovered that many lncRNAs such as *MALAT1* [282], *XIST* [283], *HOTAIR* [284], *GAS5* [285], or *DANCR* [286] contain one to several m6A modifications, and thus deoxyribozymes could be used as therapeutics for OC [287].

Gene-editing methods like zinc finger nucleases (ZFNs), transcription activator-like effector nucleases (TALENs), and clustered regularly interspaced short palindromic repeats/CRISPR-associated protein 9 (CRISPR/Cas9) system can be used to suppress lncRNA expression [288,289]. It is worth noting that, unlike protein-coding genes, lncRNA genes are not vulnerable to small insertions, deletions, or frameshift mutations so their genes must be edited to a much larger extent. In addition, lncRNA expression can sterically be repressed by CRISPR interference (CRISPRi) [290]. CRISPRi uses guide RNA (gRNA) for recognizing the target gene and catalytically dead Cas9 (dCas9) protein without endonuclease activity for blocking initiation or elongation of transcription. Similarly, CRISPR activation (CRISPRa) can be used for sequence-specific activation of gene expression. Besides several large scales, genome-wide deletion of up to several thousand lncRNA loci [291,292], CRISPRi was successfully used for knocking out particular, single lncRNA such as *MALAT1* [293], *XIST* [294], *HOTAIR* [295], *NEAT1* [296] or *UCA1* [297]. Furthermore, instead of transcriptional silencing of the whole lncRNA gene, CRISPR/Cas9 could be also used for “repairing” alleles of lncRNAs genes found to be associated with ovarian cancer susceptibility, such as *HOTAIR* polymorphism rs920778 [298].

Manipulation with lncRNAs expression levels with any of the aforementioned methods could be used to interfere with the function of lncRNAs as miRNA sponges. Increased expression of lncRNAs that bind oncomiRs or decreased expression of lncRNAs that bind tumor-suppressor miRNAs could normalize gene regulatory network and signaling pathways, and reverse malignant phenotype, just like using miRNA mimics or antagomiRs [299]. For instance, since the expression of ceRNA *SNHG5* is reduced in paclitaxel-resistant OC patients, either *SNHG5* overexpression or miR-23a inhibition could enhance paclitaxel sensitivity [300]. Similarly, ceRNA *UCA1* can sponge miR-654-5p, while its knockdown enhances miR-654-5p expression, which reduces ovarian tumor cells viability in vitro and in vivo [301].

As mentioned above, the second general therapeutic approach for targeting lncRNAs in OC would be to abolish their function by inhibiting their interactions with other macromolecules, either through competition or steric blockade. There are several approaches including aptamers, nanobodies, small molecules, and RNA decoys [302]. Aptamers are short (up to 200 nucleotides) ss DNA or RNA molecules with high specificity and affinity for their targets and are considered nucleic acid analogs of antibodies [303]. However, they have better tissue penetration, lower immunogenicity, and in vivo stability. They act through dynamic three-dimensional structure, by recognizing the secondary structure of lncRNAs, and thus interfering with lncRNA-protein interactions [304]. For instance, there exist aptamers against *HOTAIR* [305] and *H19* [306], which could be used as OC therapeutics. Nanobodies are heavy chain-only antibodies (HCAbs), found naturally in sharks and camelids, that is built of a single variable domain (VHH), which is similar to the Fab fragment of human IgG antibodies and thus non-immunogenic [307]. They have both high affinity and specificity and the potential to interrupt lncRNA-RBP interactions [308]. Nanobodies can be designed to specifically target highly structured RNA molecules [309], and since it is known that many lncRNAs such as *MALAT1* [310], *NEAT1* [311], *XIST* [230], or *HOTAIR* [312] are well-structured, this approach could be used for treating OC types in which those lncRNAs are overexpressed. Small molecules (chemical compounds), by binding to either lncRNAs or RNA-binding proteins (RBPs), can change their secondary or tertiary structures or mask protein-binding sites on lncRNAs or lncRNA-binding domains of RBPs, and thus disrupt interactions between them [313,314]. Similar to nanobodies, highly structured parts of lncRNAs can also be targeted by small molecules. Therefore, the use of high-throughput screening identified a small molecule ellipticine that can inhibit interactions between *HOTAIR*-EZH2 and *BDNF-AS-*EZH2 [315]. Furthermore, several small molecules have been discovered that can target *MALAT1* [316,317]. RNA decoys or imitators of lncRNAs could be designed to act through binding to and sequestering of proteins, to disrupt the creation of functional lncRNA-RBP complexes [318]. One such example is an anti-HIV decoy that targets the HIV-1 Tat protein. It has a trans-activation response (TAR) element RNA hairpin and binds to the Tat protein. This decoy localizes in the nucleolus while natural TAR RNA is localized in the nucleus [319]. Another example is a mimic of *HULC*, a lncRNA that interacts with phenylalanine hydroxylase (PAH) and modulates its function. *HULC* depletion causes reduced enzymatic activities of PAH, which is a characteristic of metabolic disorder phenylketonuria. The introduction of *HULC* mimics successfully restores the function of that liver lncRNA and reduces excess phenylalanine levels [320]. Therefore, this therapeutic approach could be used for treating OC types in which reduced levels of certain lncRNA, e.g., those that were in Table 2 and Table 3 classified as a tumor suppressor, is a cause for OC initiation, tumorigenesis, or metastasis.

Regulatory regions of lncRNAs could be also used for constructing more efficient drugs. There exists a DNA plasmid called H19-DTA (BC-819) that carries the gene for A subunit of diphtheria toxin under the regulation of the *H19* promoter and it is used for treating cancers with high *H19* expression such as OC [321]. Its safety and efficacy for treating ovarian and peritoneal cancer patients with advanced recurrent disease had already been proven in the Phase I/IIa clinical trial [322].

All previously described methods consider targeting a lncRNA of interest as a primary therapeutic goal for treating OCs. However, targeting lncRNAs could also be used to enhance the efficiency of already applied therapeutic regimes, since for many OC-related lncRNAs described in this paper their involvement in resistance to radio- and chemotherapy was mentioned. This has been comprehensively reviewed in [323,324].

Although constantly on the rise in recent years [325], RNA-based or RNA-targeted therapeutic approaches still have many limitations, such as inefficient delivery to the target tissue, toxicity and immunogenicity, and off-target effects (non-selectivity). Over the past decade, in parallel with the development of novel RNA therapeutics, promising approaches have been developed to overcome these hurdles and to boost their success [326]. Hopefully, this will bring to more (successful) clinical trials for targeting long non-coding RNAs as a potential therapeutic approach for the treatment of OCs.

## 9. Online Resources for lncRNA Research

Expanding interest in lncRNA research has led to the accumulation of a vast amount of knowledge that has to be properly analyzed, organized, and made available for the wider scientific community. Therefore, a constantly growing number of web resources and bioinformatical tools have been developed that can help many different aspects of lncRNA research [327,328]. However, for an ordinary wet-lab lncRNA cancer researcher without advanced computational skills, databases, and online tools would be the most useful [329]. Unlike miRbase for microRNAs [330], there is still no primary, central repository for lncRNAs. In Table 4 there is a shortlist of the best known and most used lncRNA web resources.

## 10. Conclusions and Future Perspectives

Recently we celebrated the 20th anniversary of publishing the draft human genome sequence. Initially, only about 2% of the human genome was reported to comprise protein-coding genes, and the rest referred to as “junk” DNA, it has become increasingly evident that this “junk” DNA is a goldmine for many regulatory non-coding transcripts. One type of such transcript is the lncRNAs. Consequently, their mechanisms of action and the molecular processes which they regulate are still being unraveled. As anticipated, their causative roles in the development and progression of many human neoplasms, including OCs, are becoming increasingly evident.

The major drawback in discovering the (in)distinctive roles of lncRNAs in common and rare OCs is the lack of studies that clearly and properly separate the OC subtypes. One potential reason for that could be both clinicians’ and researchers’ inexperience in diagnosing rare gynecological cancers. On the other hand, the manuscript reviewers should insist that authors need to put more effort into describing their clinical samples. Hopefully, international collaborations such as the GYNOCARE COST Action will contribute to a more unified and standardized diagnosis and classification of ROCs [1].

Long non-coding RNA molecules are promising diagnostic and prognostic biomarkers for ROCs [353]. Currently, we have been witnessing an accelerated development and an increased efficiency of RNA-bases therapeutics. Further in-depth knowledge about lncRNAs should direct researchers and pharmaceutical companies in the post-COVID era to become more interested in lncRNAs as potential therapeutic agents or therapeutic targets. These emerging technologies and approaches could certainly improve the quality of life and outcome of many women with ROCs.

## Figures and Tables

**Table 4:** A short overview of online resources useful for lncRNA research in cancer.

Type	Web Address	Reference
General lncRNA Databases		
RNAcentral	https://rnacentral.org/	[331]
LNCipedia	https://lncipedia.org/	[332]
LncBook	http://bigd.big.ac.cn/lncbook/index	[333]
lncRNAdb	http://lncrnadb.org/	[334]
lncRNome	http://genome.igib.res.in/lncRNome/	[335]
GENCODE	https://www.gencodegenes.org/	[55]
General Expression Databases		
GTEx	https://gtexportal.org/home/	[336]
GEO	https://www.ncbi.nlm.nih.gov/geo/	[337]
TCGA	https://portal.gdc.cancer.gov/	[338]
Expression Atlas	https://www.ebi.ac.uk/gxa/home	[339]
The lncRNA-Specific Expression Databases		
NONCODEV5	http://v5.noncode.org/index.php	[340]
NRED	http://jsm-research.imb.uq.edu.au/nred/cgi-bin/ncrnadb.pl	[341]
LncExpDB	https://bigd.big.ac.cn/lncexpdb/	[342]
LncSpA	http://bio-bigdata.hrbmu.edu.cn/LncSpA/	[343]
General Disease- and Cancer-Specific lncRNA Databases		
TANRIC	https://www.tanric.org/	[344]
LncRNADisease 2.0	http://www.rnanut.net/lncrnadisease/	[345]
Lnc2Cancer 3.0	http://www.bio-bigdata.com/lnc2cancer/	[346]
lncRNASNP2	http://bioinfo.life.hust.edu.cn/lncRNASNP/	[347]
Lnc2Catlas	https://lnc2catlas.bioinfotech.org/	[348]
Function-Specific and Other Useful lncRNA Databases		
DIANA-LncBase v3	https://diana.e-ce.uth.gr/lncbasev3/home	[349]
SEEKR	http://seekr.org/home	[350]
LPI-MiRNA	https://github.com/zyk2118216069/LncRNA-protein-interactions-prediction	[351]
LncRNAWiki	http://lncrna.big.ac.cn	[352]
lncRNA Blog	https://www.lncrnablog.com/	-

## Abbreviations

BEP	bleomycin, etoposide, and cisplatin
CAF	cancer-associated fibroblasts
ceRNA	competing endogenous RNA
CSC	cancer stem cells
EC	endometroid carcinoma
EMT	epithelial to mesenchymal transition
EOC	epithelial ovarian cancer
FIGO	Fédération Internationale de Gynécologie et d’Obstétrique
GCT	granulosa cell tumor
HGSOC	high-grade serous ovarian cancer
HOTAIR	HOX transcript antisense RNA
IOT	immature ovarian teratocarcinoma
LGSOC	low-grade serous ovarian cancer
lincRNA	long intergenic non-coding RNA
lncRNA	long non-coding RNA
MALAT1	metastasis-associated lung adenocarcinoma transcript 1
MSC	mesenchymal stem cell
MET	mesenchymal to epithelial transition
miRNA/miR	microRNA
MOC	mucinous ovarian carcinoma
NAT	natural antisense transcript
ncRNA	non-coding RNA
OC	ovarian cancer
OCCC	ovarian clear cell carcinoma
OCSC	ovarian cancer stem cells
PCOS	polycystic ovary syndrome
PRC	Polycomb Repressive Complex
RNAi	RNA interference
ROC	rare ovarian cancer
siRNA	small interfering RNA
shRNA	short hairpin RNA
XIST	X-inactive specific transcript
